# Correlation between Family *RB1* Gene Pathogenic Variant with Clinical Features and Prognosis of Retinoblastoma under 5 Years Old

**DOI:** 10.1155/2021/9981028

**Published:** 2021-07-12

**Authors:** Yi Zhang, Yizhuo Wang, Dongsheng Huang, Jianmin Ma, Weiling Zhang, Huali Gu, Yan Zhou, You Yi, Pinwei Zhang

**Affiliations:** ^1^Department of Pediatrics, Beijing Tongren Hospital, Capital Medical University, No 2, Xihuan South Road, Yizhuang Economic and Technological Development Zone, Beijing 100176, China; ^2^Beijing Tongren Eye Center, Beijing Tongren Hospital, Capital Medical University, Beijing Institute of Ophthalmology, Beijing Key Laboratory of Ophthalmology and Visual Sciences, Beijing 100730, China

## Abstract

Retinoblastoma (RB) is the most common primary intraocular malignant tumor in infants and the prototype of human hereditary tumors. Its occurrence and development are closely related to the pathogenic variant of tumor suppressor RB1 gene. We aim to analyze the characteristics of RB1 gene pathogenic variant and clinical phenotype in retinoblastoma patients and their relatives. Children with RB were recruited from August 2007 to November 2017. QT-PCR, probing, and gene sequencing were used to analyze the sequence of *RB1* gene in RB children, their parents, or grandparents with a clear history of illness. The SPSS20.0 software was used to analyze the correlation between polymorphisms of *RB1* gene and the incidence and prognosis of the enrolled children and relatives. 40 RB children (20 males and 20 females) were recruited, unilateral RB accounted for 52.5% (21/40), bilateral RB accounted for 42.5% (17/40), and trilateral RB accounted for 5.0% (2/40). 6 patients had a clear family history (15.0%, 6/40). It had been verified that 19 probands (47.5%) have *RB1* gene pathogenic variants (11 frameshift and 8 missense pathogenic variants), of which germline inheritance accounted for 47.4% (9/19) and nongermline heredity accounted for 52.6% (10/19). Pathogenic variants of 10 nucleic acid sites without reported were found, among which c.2455C>G (p.L819V) was confirmed to have heterozygous pathogenic variants in both a bilateral RB patient and his mother with unilateral RB. Family genetic high-risk factors, bilateral/trilateral RB, >12-month-onset RB have a higher proportion of *RB1* gene pathogenic variant than children with no family history, unilateral RB, and ≤12-month (*P* = 0.021, 0.001,0.034). The proportion of pedigree inheritance of infantile retinoblastoma with bilateral disease is high. There was a certain proportion of *RB1* gene pathogenic variant in 3-5-year-old children with bilateral RB, even if they had no family genetic history. Therefore, the detection of *RB1* gene pathogenic variant should not only focus on infants but also on the phenotype of *RB1* gene pathogenic variant in children over 3 years old with bilateral eye disease.

## 1. Introduction

Retinoblastoma (RB) is the most common primary intraocular malignant tumor in infants and the prototype of human hereditary tumors. RB could be divided into two genetic patterns of germline inheritance: germline inheritance and nongermline inheritance [1]. The germline inheritance is mainly germ cell pathogenic variant, presenting with bilateral RB (account for about 20-30%) or unilateral multiple RB (account for about 70-80%), of which about 5% can be accompanied by intracranial tumors, such as pineal gland tumor and primary neuroblastoma on or near the sella turcica, which is called trilateral RB [2]. Nongermline inheritance is caused by somatic pathogenic variant, mostly unilateral Rb. Of all RB, genetic RB account for about 45%, nongenetic RB account for 55%, and about 5%-10% of children have a clear family history [1, 3–6]. The occurrence and development of RB are closely related to the pathogenic variant of the tumor suppressor RB1 gene [7]. Since the 1980s, the *RB1* gene on the long arm of chromosome 13 (13q14) has been recognized as the RB tumor suppressor gene for the first time, and its pathogenic variant is involved in the occurrence of RB [5, 6, 8–10]. Compelling studies [1, 5, 6, 11–15] indicated that the *RB1* gene is an allele encoding retinoblastoma protein (pRB), and the lack of monitoring or inactivation of *RB1* will lead to a decline in cell proliferation regulation function thus resulting in abnormal cell proliferation.

At present, the use of PCR, first-generation sequencing technology, high-throughput sequencing, and probe technology could detect *RB1* gene pathogenic variant, which provides a useful reference for prognosis evaluation, treatment planning, and family planning consultation [5, 12–14]. However, gene pathogenic variant often results in the failure of protein translation and synthesis due to the nucleic acid or base pathogenic variant in the coding region involved in protein translation and transcription. The detection of chromosome 13 and the pathogenic variant of the *RB1* gene are well reported [5], but the mechanism of the analysis of the nucleic acid polymorphism of the coding region on the pathogenesis and efficacy of RB is relatively rare.

Since 2005, our hospital has collected more than 2,000 retinoblastoma cases; however, the proportion of children with clear family history is less than 3%, and some children with unilateral eye disease appeared heterogeneous binocular disease. The detection and analysis of *RB1* gene pathogenic variant may be helpful for the early development and early warning of disease progression. Due to the late application of genetic technology, the study on the relation between clinical therapy and the detection of polymorphisms in the familial *RB1* gene is rare. In this study, we collected 40 cases of pathological/clinically diagnosed children with RB. The *RB1* gene polymorphism was measured in these children and their parents. For children with a clear family history, their maternal grandparents were simultaneously analyzed with informed consent. Combined with their clinical data, we analyze the impact of *RB1* gene polymorphism on morbidity of RB and provide a molecular level diagnosis information.

## 2. Patients and Methods

### 2.1. Patients

In this retrospective analysis, the diagnosis and treatment procedure have been approved by the ethics committee (TRECKY2019-034). From August 2007 to November 2017, a series of cases were included. The informed consent was obtained from the legal guardian of the patients. The inclusion criteria were as follows: (1) the sample is collected before the chemotherapy treatment, (2) male or female patients with the first onset age of <5 years (60 months), (3) RB was initial diagnosed histopathological and initial treated in our hospital, and (4) complete follow-up data and informed consent with regular treatment and follow-up. Rejection or withdrawal criteria: patients who did not meet one of the above criteria are rejected or withdrawn.

### 2.2. Diagnostic Criteria [16–18]

The clinical diagnosis was based on transocular ultrasonography, and Retcam machine fundus examination and orbital CT/MRI suggesting that the occupancy of the lesion is within the sphere and calcification is visible. The diagnosis was confirmed to be consistent with the diagnosis of RB by histopathology and immunohistochemistry. According to the international classification of retinoblastoma (ICRB) in 2003 [19], RB was classified into I-IV stages. According to the IIRC system, the intraocular stage was divided into A-E groups for the serious classification. Other imaging examinations including fundus examination under general anesthesia by Retcam, whole-body bone scan combined chest and abdominal enhanced pelvis CT. Patients were divided into 4 groups according to risk factors: low-risk group (no high-risk factors), intermedium-risk group (with 1 high-risk factor), high-risk group (infringement of optic nerve ends, or 2 or more high-risk factors), and ultrahigh-risk group (with intracranial metastasis or distant metastasis).

### 2.3. Samples

A total of 5 ml whole blood samples were drawn from each child into EDTA tubes before treatment. 10 ml of whole blood from the relatives of the child was taken on fasting. Phenol chloroform method was used to extract the whole DNA (QLA DNA blood mini kit, Qiagen). First, add 3-5 ml of whole blood into a 50 ml centrifuge tube; add 30-40 ml of ddH_2_O; shake for 20 seconds; stand for 10 minutes, 4 degrees, 3800 rpm, 20 min; and remove the supernatant carefully. Next, 5 ml TES were added to the precipitation to mix it upside down (vortex oscillation, breaking cell membrane). Then, 350 *μ*l, 10% SDS, and 70 *μ*l (10 ng/*μ*l) proteinase K were added at 37°C overnight. The next day, the centrifuge tube was cooled to room temperature, 5 ml of Tris-saturated phenol was first added and mixed upside down. After that, 5 ml of isoamyl chloroform (24 : 1) was added (must be done, otherwise, it will affect the final stratification and is not conducive to the extraction of supernatant), followed by upside down mixing, 4 degrees, 2500 rpm, and centrifugation for 15 min. Take all the supernatant into another centrifuge tube, add 2.5 times volume of ice-free ethanol, repeatedly reverse, precipitate out the DNA blob directly into the 1.5 ml centrifuge tube with pipette, wash it twice with 75% ethanol, and add 200 *μ*l or 300 *μ*l after drying dissolve TB, put it 4°C later to measure OD, place the original solution at -20°C or -80°C for storage. The concentration of the extracted DNA sample was 20 ng/dl. The primers and sequence analysis of the *RB1* gene for amplification were designed by Joy Orient Translational Medicine Research Center Co., Ltd. China. Primer sequence: F-3′-GTAACCAGGTCATGTAGCA-5′; R-3′-GCCTTAGGTAGACGGATC-5′.

### 2.4. High-Throughput Sequencing of *RB1* Gene

Online database (https://www.ncbi.nlm.nih.gov/assembly/CF_000001405.39/) indicates the total length of the *RB1* gene (13q14.2) sequence is 3,099,706,404 bp, 28 exons, and some adjacent introns. A nucleic acid analysis (Agilent Sureselect Target Enrichment human-exome system) was used for *RB1* gene analysis. The DNA sequence analysis is based on the GATK standard establishment algorithm (GATK V.4, version 2.0). To detect the pathogenic variants, deletions, and insertions of nucleic acid sites in the *RB1* gene, probe technology, fluorescence electrophoresis, QT-PCR (Applied Biosystems Foster City, CA, USA), and fragment sequencing were used for verification. All mutation sites will be checked online based on 1000 Genomes data (http://http://www.1000.genomes.org/), nucleic acid, and base mutations (http://www.ncbi.nih.gov/projects/snp/). Mutation on exons was represented by c.-xxx, and introns are represented by IVS+xxx. Pathogenic variant type includes base insertion (Ins) and deletion (del). The base length unit is bp. Undefined germline genetic pathogenic variant of *RB1* gene refers to the frameshift or missense pathogenic variant of nucleic acid or base point in the coding region of *RB1* gene, but it has not been confirmed by online database or domestic and foreign harmful pathogenic variants.

### 2.5. Determination of Genetic Type [1, 4–6]

(1) Germline inheritance could be confirmed if direct family members clearly have RB patients with or without *RB1* locus variation; *RB1* gene has locus variation, and the same locus variation exists in relatives. (2) Nongermline inheritance: only the proband's *RB1* gene site pathogenic variant exists, and there are no high-risk relatives and genetic site pathogenic variants

### 2.6. Statistical Analysis

The SPSS 20.0 software was used for analysis. Statistical description and frequency description were used for quantitative data. The measurement data of normal distribution is (average ± standard deviation), and that of nonnormal distribution is M (Q1, Q3). The count data is expressed as the composition ratio or percentage (%). Kaplan Meier curve was used for survival analysis, and Cox regression was used to analyze the influence of risk factors on prognosis. Fisher's precise inspection was used for statistical analysis. *P* < 0.05 was considered as a statistical difference.

## 3. Results

### 3.1. Demographic Data

**Figure 1:**
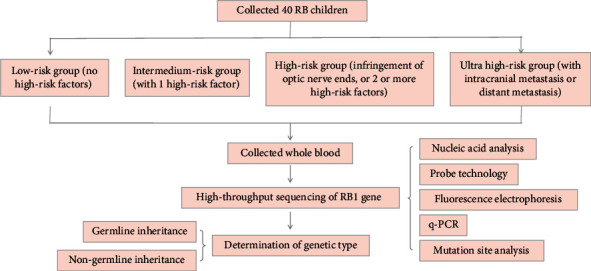
Schematic representation of the study.

40 patients were enrolled with 20 males and 20 females. Monocular disease occurred in 21 cases (52.5%) and 17 cases (42.5%) with bilateral eyes. The other 2 cases (5.0%) with trilateral RB and the number of disease eyes were 61. The median age of these children was 15.5 months (1-53 months) (Figure 1 and Table 1). Among the 40 children, 6 had a family history (15.0%), and 34 had no clear family history (85.0%). After diagnosis, ophthalmectomy (removal of heavy eyes in children with two eyes) was performed in 23 cases, and 2 cases of RB patients with trilateral eyes were classified as grade IV due to pineal gland occupancy. The composition of pathology grade was grade I accounted for 48% (12/25), grade II accounts for 16.0% (4/25), grade III accounts for 24.0% (6/25), and grade IV accounts for 12.0% (3/25). Vitrectomy was performed in 2 cases; 3 patients received interventional therapy; 6 patients underwent sheath injection according to pathological grading. 39 patients received chemotherapy. The median chemotherapy cycle was 6 cycles (2-17 cycles).

### 3.2. *RB1* Gene Analysis

Mutations, insertions, deletions, and other site pathogenic variants of *RB1* gene occurred in 19 of 40 patients (47.5%), of which 11 were frameshift pathogenic variants (57.9%,11/19), and of which 8 were inserted missense pathogenic variants (42.1%,8/19); no pathogenic variants were found in 21 cases of children (52.5%). Combined with family history and *RB1* gene phenotype, 9 cases of RB were germline inheritance in genetic type (22.5%, 9/40), 10 cases were *RB1* gene site pathogenic variants (25.0%, 10/40), and the remaining 21 cases had no evidence of genetic tendency (52.5%, 21/40). Fisher's clinical analysis suggested that for children with germline inheritance undergo eyeball removal, the ratio of postoperative tumor invasion to the posterior bulb optic nerve/end was higher than that of children with only tumor gene site pathogenic variants (*P* = 0.025). Among the 9 cases of germline inheritance, 6 cases have a clear family history. Three children and relatives have the same pathogenic variant site. 21 children and their relatives had no pathogenic variant phenotype (52.5%), of which 1 patient with binocular disease had a family genetic history, 1 patient had congenital motor dysplasia, and the remaining 19 patients had no family genetic and high-risk factors for congenital diseases (Tables 1 and 2). We found 10 probands with novel genetic variants: c.2124_2125insT, c.2455C>G, IVS1+372G>C, c.1686_1688delATG, c.2092A>T, IVS18+1_47del47, c.376_380; IVS3+1_25delinsGGAAACGAATT, c.425_428delCCAA, c.62delC, IVS4+50T>C, Table 3.

As we can see from Table 3 (case 2), the proband and the mother of the proband simultaneously had a heterozygous pathogenic variant at position c.2455C>G (p.L819V). In Table 3 (case 8), the proband had a missense pathogenic variant in base deletion. Although the *RB1* gene pathogenic variant was not detected in relatives, the mother was a patient. The proportion of *RB1* gene site pathogenic variant is higher in those with family genetic high-risk factors, binocular/trilateral RB, >12-month-onset RB, and in the high-risk group than those with no family history, unilateral eye RB, ≤12-month-old and low-risk group (*P* = 0.021, 0.001, 0.034, 0.049, respectively) Table 4. The pathogenic variant was correlated with pathological grade after eyeball removal with significant difference (*R* = 4.753, *P* = 0.029).

### 3.3. *RB1* Gene Pathogenic Variants and Diagnosis of Age Feature

In 13 RB cases aged <12 months old, only 4 bilateral RB cases had *RB1* gene pathogenic variant (one of frameshift pathogenic variants, two of excluding deletion or loss of heterozygosity). According to family history and kindred phenotype, the genetic type of 3 bilateral RB was germline inheritance, and only one bilateral RB patient's genetic type was gene site mutation. The difference of pathogenic variant type in infant bilateral RB cases was significant (*P* = 0.049) (Tables 5 and 6). Other 4 bilateral RB and 6 unilateral RB had no *RB1* gene pathogenic variant. Although the proportion of *RB1* gene pathogenic variant in bilateral disease was higher than unilateral cases, statistical analysis showed that there was no significant difference in *RB1* gene pathogenic variant between <12 months old children (*P* = 0.122) (Tables 5 and 6).

In 27 RB cases aged >12months old (23 cases were 12-36months old and 4 cases were 36-60 months old), *RB1* gene pathogenic variants were found in 16 of 27 children (59.3%), including 10 of frameshift pathogenic variants or excluding deletion or loss of heterozygosity (62.5%, 10/16), and 6 cases were germline inheritance (2 unilateral RB cases and 4 bilateral/trilateral RB cases) (37.5%, 6/16), as shown in Tables 6 and 7. Statistical analysis showed that the proportion of *RB1* gene pathogenic variant in bilateral and/or trilateral RB was significantly higher than that of unilateral RB (*P* = 0.003) (Table 5). In 23 patients aged 12-36 months old, the proportion of *RB1* gene pathogenic variants in bilateral eyes/trilateral eyes (88.9%, 8/9) was higher than that in unilateral eyes (25.7%, 5/14). The pathogenic variant type was mainly *RB1* gene site mutation. The differences were all significant (*P* = 0.027, 0.025) (Tables 6 and 7). In 4 RB patients aged 36-60 months old, 2 bilateral RB cases and 1 trilateral RB case had gene mutation, and only 1 case of unilateral eye RB had no gene mutation.

## 4. Discussion

Approximately 9,000 newborn babies are diagnosed with RB every year. Most of RB onset within 3 years of age without gender differences, while about 3000 children die of RB every year [20, 21]. Canada reported that the average age of diagnosis of monocular onset is 27 months, the average age of diagnosis of binocular onset is 15 months, and the average age of diagnosis of single and double eyes in Kenya is 36 months and 25 months [1, 3, 6]. In our retrospective study, there were 20 males and 20 females, and RB onset within 5 years of age, with a median age of 15.5 months, which basically consistent with the literature reports [1, 3, 22].

RB cells are derived from susceptible vertebral photoreceptor cells of retinal precursors. When alleles of the *RB1* gene are mutated or deleted, RB cells could remain in the inner core layer, initially act as benign precursor “retinoma,” with gene pathogenic variant increased, aggregation and uncontrolled cell proliferation lead to retinoblastoma [15, 16]. At present, RB gene detection is widely used in screening and detection of *RB1* mutation carriers in patients' relatives, as well as prenatal testing [23]. In this study, 47.5% (19/40) of children with RB had *RB1* gene pathogenic variants, of which 22.5% (9/40) had germline inheritance (6 cases had a clear family history of onset, 3 cases children and relatives had the same mutated and displayed locus), which is basically consistent with the above report. It is worth noting that one patient and his mother are both RB patients, and the child is binocular and the mother is monocular RB. However, the *RB1* gene test showed no harmful mutations, indicating that even without genetic mutations, family genetic risk is still one of the characteristics of germline inheritance, and the pathogenesis of molecular biology should be further studied.

In this group, the incidence of germline genetic and tumor gene pathogenic variant was higher in binocular RB with significant difference (*P* = 0.002). In this group of cases, 2 cases of trilateral RB had gene pathogenic variants; The proportion of *RB1* gene site pathogenic variant in children with family genetic risk factors was significantly higher than that in children without family genetic risk factors (*P* = 0.021); there was also a difference between age factors and *RB1* gene pathogenic variants. Chai et al. found that *RB1*-mutated patients presented with earlier age of diagnosis, with a significantly larger proportion of bilateral cases and secondary malignancies relative to those without *RB1* mutations [24]. To some extent, our results were consistent with those of Chai et al. Therefore, it indicated that the pathogenic variant of the *RB1* gene was closely related to the onset characteristics. We also observed that the proportion of *RB1* gene pathogenic variant in children >12 months was higher than that in infants (59.3% vs. 16.7%, *P* = 0.034), which was different from previous literature reports and should be further analyzed of its pathogenesis, which might provide a basis for future diagnosis and treatment. Although the pathogenic variant rate of *RB1* gene in infant RB was lower than that in RB children aged >12 months, the pathogenic variant rate of infants with bilateral RB was relatively high, and it was mainly inherited by species, from the results of our study. In our study, the cause of some unilateral RB in aged >12 months was related to *RB1* gene site mutation, although without family genetic factors, which might lead to tumor after the second hit of RB cells reported in previous reports [1, 5, 6]. The proportion of *RB1* gene pathogenic variant in RB of bilateral/trilateral eyes was significantly increased in this group, including the older age group. *RB1* gene pathogenic variant was not limited to children with previous infantile onset, while familial genetic factors were not dominant in children over 12 months old. Therefore, we suggested that *RB1* gene pathogenic variant screening should be paid attention to in children with bilateral and/or trilateral RB under 5 years old, so as to do a good job in genetic counseling.

Identification of the *RB1* gene mutation and the genetic pattern was helpful to strengthen the clinical management of high-risk relatives [25]. RB gene mutation analysis can help nurses to improve the management of RB patients from different aspects, guide the screening plan for some high-risk relatives of RB patients with positive *RB1* mutation detection, and eliminate unnecessary screening for relatives without disease risk. From the analysis of *RB1* gene pathogenic variant types, frameshift pathogenic variants accounted for 57.9% (11/19), and base-missing/insertion missense pathogenic variants accounted for 42.1% (8/19). However, in nongermline inherence, base deletion/insertion accounts for 60%, indicating that for genetic *RB1* frameshift pathogenic variants are mostly occurred to transmit genetic information, while for nongermline inheritance, due to tumor gene pathogenic variants mostly occurred, their own missense pathogenic variants become the main cause of disease. Due to the lack of RB protein, nonsense and frameshift mutations often lead to bilateral multifocal tumors [26]. In addition, our study found that 10 undefined pathogenic variant sites existed in 10 children, while it needs further verification to identify whether it is a clear harmful pathogenic variant. As we can see from Table 3, there is a heterozygous pathogenic variant at position c.2455C>G (p.L819V) in the proband (case 2) and his mother at the same time. Therefore, this pathogenic variant has not been reported, and it should be concerned. In case 8 of Table 3, the child has a missense pathogenic variant in base deletion. Although the mother was not detected to have the *RB1* gene pathogenic variant, she still had a unilateral eye disease. Therefore, it should be noted that the genetic mutation of the offspring may be caused by genetically high-risk factors.

The treatment of RB should be based on the primary purpose of saving life, and a variety of treatment methods [27, 28] should be adapted. The current treatment regimens were based on a variety of different factors, such as clinical staging at initial diagnosis, intraocular staging, onset eyes, family genetics, and family decisions, including conservative treatment (intervention, glass group removal, laser, and freezing), surgical resection including eyeball removal and orbital content removal, and systemic chemotherapy and radiotherapy treatment. For distant metastasis, stem cell transplantation could also be used in children at this stage [20, 21, 29]. At present, the overall survival rate in developed countries and China has reached more than 90%; however, the mortality rate in backward countries such as Kenya is still high [1, 3, 6]. This may cause by the lack of timely diagnosis and treatment.

Although the children in this group mainly developed RB in the intraocular period, only 2 cases were a special type of RB in the saddle area and the pituitary, while they still mainly in the D and E stages. Therefore, systemic chemotherapy combined with radiotherapy and sheath injection therapy was mainly used, and local treatment was combined in some children. Prognostic analysis suggests that the removal of the eyeball, the presence of recurrence, and the use of sheath injection therapy in children after posterior optic nerve invasion are all risk factors that affect the prognosis, regardless of whether there is a family history or genetic pathogenic variant. The above results showed that the prognosis of RB was affected by many factors, especially the treatment. Although the patients with *RB1* gene pathogenic variant are relatively complex, reasonable treatment can still achieve a better long-term survival. But our research is still insufficient, most of the patients in our clinic were sporadic cases, so our series included sporadic cases and a few familial cases. However, we believe that our findings may help to facilitate further large-scale studies to investigate this correlation.

In conclusion, the mutation of the *RB1* gene and the presence of family genetic risk factor are closely related to the occurrence of RB disease, and the prognosis should also be combined with multiple factors such as patient's tolerance to the treatment, diagnosis, and treatment methods. Pathogenic variants of 10 nucleic acid sites without reported were found in this study, among which c.2455C>G (p.L819V) was confirmed to have heterozygous mutations in a bilateral RB patient and his mother with unilateral RB. The proportion of pedigree inheritance of infantile retinoblastoma with bilateral disease was higher. There was a certain proportion of RB1 gene mutation in 3-5-year-old children with bilateral RB, even if they had no family genetic history. Therefore, the detection of the absence or pathogenic variant of the *RB1* gene has positive significance for clinical diagnosis and treatment, and we suggest that the detection of *RB1* gene mutation should not only focus on infants but also on the phenotype of *RB1* gene mutation in children over 3 years old with bilateral eye disease.

## Figures and Tables

**Table 1:** Demographic data of the 40 children.

Category	*N* (%)
Age	40
0 ~ 12 months	13 (32.5%)
>12 months	27 (67.5%)
12-36 months	23 (85.2%)
36-60 months	4 (14.8%)
Family genetic history	40
With family history	6 (15%)
Bilateral RB	5
Trilateral RB	1
Without family history	34 (85%)
Unilateral RB	21
Bilateral RB	12
Trilateral RB	1
Treatment	40
Interventional therapy+chemotherapy+intrathecal injection	13 (32.5%)
Chemotherapy±intrathecal injection	2 (5%)
Eyeball removal+chemotherapy±intrathecal injection	22 (55%)
Eyeball removal+vitrectomy+chemotherapy±interventional therapy±intrathecal injection	1 (2.5%)
Chemotherapy+vitrectomy	1 (2.5%)
Risk grouping	40
LR	29 (72.5%)
IR	6 (15.0%)
HR	5 (12.5%)
Prognosis	40
Survival	31 (77.5%)
Dead	6 (15.0%)
Lost follow-up	3 (7.5%)
Onset eye and genetic type	40
Unilateral RB	21
Germline inheritance/tumor gene pathogenic variant/no genetic characteristics	2/3/16
Bilateral RB	17
Germline inheritance/tumor gene pathogenic variant/no genetic characteristics	6/6/5
Trilateral RB	2
Germline inheritance/tumor gene pathogenic variant	1/1
Genotype	19
Germline inheritance^∗^	9
Frameshift pathogenic variant/base deletion or insertion/no pathogenic variant	7/1/1
Nongermline inheritance	10
Frameshift pathogenic variant/base deletion or insertion	4/6

^∗^1 case with a clear family history proband and diseased relative (mother) RB1 gene has no harmful pathogenic variants; ^#^, no children underwent binocular removal.

**Table 2:** Clinical data of 9 children with germline genetic phenotype.

Case	Onset age (month)	Eye classification	Pathological grade	c.DNA change	Amino acid change	Proband phenotype	Kinship phenotype	Family genetic risk factors	Prognosis
1	11	Bilateral	II	No mutation	—	No mutation	No mutation	Mother was patient	Dead
2	15	Bilateral	—	c.1735C>T	p.R579X	TC	CC (maternal)	Mother's monocular onset	Dead
3	2	Bilateral	I	IVS24+1G>T	—	TT	TG (maternal) TG (maternal grandfather)	Grandmother had unilateral eye disease	EFS
4	2	Bilateral	—	c.1333C>T	p.R445X	TC	TC (maternal) TC (maternal grandmother)	Unilateral eye RB of grandmother and her mother	EFS
5	12	Bilateral		c.425_428delCCAA	—	CCAA del	CCAA	Brother died of RB	Survival with tumor
6	2	Trilateral	IV	c.2664-10T>A	—	AT	TT (parent)	Congenital hydrocephalus of child	Dead
7	17	Unilateral	I	c.2455C>G	p.L819V	GC	GC (maternal)	—	EFS
8	2	Unilateral	I	c.6249C>G c.6119G>A	p.I2083M p.R2040Q	GC/AG	CC/AG (paternal) GC/GG (maternal)	—	EFS
9	26	Bilateral	I	c.1531_1532GG>AA	—	AG/AG	GG/AG (parent)	—	EFS

**Table 3:** Undefined *RB1* gene pathogenic variant site and clinical data.

Case	Onset age (month)	IIRC staging∗	Pathological grade	cDNA change	Amino acid change	Proband phenotype	Family history	Kinship phenotype is consistent or not	Prognosis
1	53	D/E	IV	c.2124_2125insT	p.Y709Lfs^∗^12	T ins	No	No	Dead
2	17	E/	III	c.2455C>G	p.L819V	GC	No	Yes (mother)	Survival
3	15	D/E	I	IVS1+372G>C	—	CG	No	No	Survival
4	3	E/B	I	c.1686_1688del ATG	p.W563del	del ATG	No	No	Survival
5	3	D/D	—	c.2092A>T	p.R698W	TA	No	No	Lost follow up
6	16	D/D	—	IVS18+1_47delGTAAGCAAAATATATGTTATGTTGACCATTCAAACTGCAAATAGATT	—	GTAAGCAAAATATATGTTATGTTGACCATTCAAACTGCAAATAGATT del	No	No	Survival
7	17	D/D	—	c.376_380delATCAG IVS3+1_25delGTAAAGTTTCTTGTATAAATATAAG c.376_386insGGAAACGAATT	—	ATCAGTAAAGTTTCTTGTATAAATATAAGdel GGAAACGAATT ins	No	No	Survival
8	12	D/E	—	c.425_428delCCAA	—	CCAA del	Yes	No	Survival
9	16	/E	III	c.62delC	—	C del	No	No	Survival
10	20	D/	II	IVS4+50T>C	—	CT	No	No	Survival

^∗^, X/X: left eye staging/right eye staging.

**Table 4:** Analysis of *RB1* gene pathogenic variants and onset characteristics.

Onset characteristics	*N*	*RB1* gene pathogenic variant cases (m)	% (m/n)	Statistics value (*X*^2^)	*P*
Age (mon)	40	19	47.5 (19/40)	4.607	0.019
0 ~ 12	13	3	18.6 (3/13)		
12-36	23	13	56.5 (13/23)		
4	4	3	75.0 (3/4)		
Eye classification	40	19	47.5 (19/40)	10.119	0.002
Unilateral RB	21	5	23.8 (5/21)		
Bilateral RB/trilaterall RB^∗^	19	14	76.2 (14/17)		
Risk group	40	19	47.5 (19/40)	6.278	0.049
Low-risk (LR) group	29	12	41.4 (12/29)		
Intermediate-risk (IR) group	6	2	33.3 (2/6)		
High-risk group (HR)	5	5	100.0 (5/5)		
Family genetic high-risk factors	40	19	47.5 (19/40)	5.647	0.021
Yes	10	8	80.0 (8/10)		
No	30	11	36.7 (11/30)		
Congenital developmental defects	40	19	47.5 (19/40)	0.005	0.731
Yes	2	1	50.0 (1/2)		
No	38	18	47.4 (18/38)		
Pathology grade of children with eye removal	23	9	39.1 (9/23)	0.059	0.582
Posterior optic nerve un infringed	16	6	37.5 (6/16)		
Invasion of the posterior optic nerve and end of bulb	7	3	14.3 (3/7)		

^∗^: 2 of 40 cases were trilateral RB and all had *RB1* gene pathogenic variant.

**Table 5:** *RB1* gene pathogenic variants and diagnosis of age feature in 40 RB patients.

Age	N	*RB1* gene pathogenic variant		Non-*RB1* gene pathogenic variant		*P*
		Unilateral RB (% m/n)	Bilateral RB (% m/n)	Unilateral RB (% m/n)	Bilateral RB (% m/n)	
<12 months	13	0% (0/13)	23.1% (3/13)	46.1% (6/13)	30.8% (4/13)	0.122
>12 months	27	18.5% (5/27)	40.7% (11/27)^∗^	37.0% (10/27)	3.8% (1/27)	0.003

^∗^: two patients were trilateral RB.

**Table 6:** *RB1* gene pathogenic variants and diagnosis of age and onset eye features in 40 RB patients.

Age group (months)	Number (% m/n)	*RB1* gene pathogenic variant (% m/n)	Non-*RB1* gene pathogenic variant (% m/n)	*P*
0-12 m	13 (32.5% 13/40)	23.1 (3/13)	76.9 (10/13)	0.122
Unilateral RB	6 (46.2% 6/13)	0.0 (0/6)	100.0 (6/6)	
Bilateral/trilateral RB	7 (46.13% 7/13)	42.9 (3/7)	57.1 (4/7)	
12-36 m	23 (57.5% 13/40)	56.5 (13/23)	43.5 (10/23)	0.027
Unilateral RB	14 (60.9% 14/23)	35.7 (5/14)	64.3 (9/14)	
Bilateral/trilateral RB	9 (39.1% 9/23)	88.9 (8/9)	11.1 (1/9)	
36-60 m	4 (10.0% 4/40)	75.0 (3/4)	25.0 (1/4)	0.250
Unilateral RB	1 (25.0% 1/4)	0.0 (0/1)	100.0 (1/1)	
Bilateral/trilateral RB	3 (75.0% 3/4)	100.0 (3/3)	0.0 (0/3)	

**Table 7:** Types and RB1 genetic analysis of diagnosis of age and onset eye features in 40 RB patients.

Age group (months)	Number (% m/n)	Germline inheritance (% m/n)	*RB1* gene pathogenic variant (% m/n)	Non-*RB1* gene pathogenic variant (% m/n)	*P*
0-12 m	13 (32.5% 13/40)	23.1 (3/13)	7.7 (1/13)	69.2 (9/13)	0.049
Unilateral RB	6 (46.2% 6/13)	0.0 (0/6)	0.0 (0/6)	100.0 (6/6)	
Bilateral/trilateral RB	7 (46.13% 7/13)	42.9 (3/7)	14.3 (1/7)	42.8 (3/7)	
12-36 m	23 (57.5% 13/40)	26.1 (6/23)	30.4 (7/23)	43.5 (10/23)	0.025
Unilateral RB	14 (60.9% 14/23)	14.3 (2/14)	21.4 (3/14)	64.3 (9/14)	
Bilateral/trilateral RB	9 (39.1% 9/23)	44.4 (4/9)	44.4 (4/9)	11.2 (1/9)	
36-60 m	4 (10.0% 4/40)	0.0 (0/4)	75.0 (3/4)	25.0 (1/4)	0.250
Unilateral RB	1 (25.0% 1/4)	0.0 (0/1)	0.0 (0/1)	100.0 (1/1)	
Bilateral/trilateral RB	3 (75.0% 3/4)	0.0 (0/3)	100.0 (3/3)	0.0 (0/3)	

## Data Availability

The data in this manuscript are available from the corresponding author upon request.
